# Multifunctional farming as successful pathway for the next generation of Thai farmers

**DOI:** 10.1371/journal.pone.0267351

**Published:** 2022-04-25

**Authors:** Para Jansuwan, Kerstin K. Zander

**Affiliations:** 1 Northern Institute, Charles Darwin University, Darwin, NT, Australia; 2 Centre for Project and Programme Evaluation, Office of Agricultural Economics, Bangkok, Thailand; Gebze Teknik Universitesi, TURKEY

## Abstract

Young farmers play a significant role in sustaining food security and the communities’ and rural areas’ viability. However, as with many countries, Thailand is facing a decline in the number of young farmers who, if not productive and satisfied with their farm business, are likely to exit farming to take advantage of their increased educational level and off-farm job opportunities. Data were collected by interviewing young farmers in the Prachin Buri province, Thailand, with the aim of assessing their reason for farming in the long-term and the type of farming. Farming decisions can be categorised into three types: full-time profit-oriented farming with a focus on rice production (~53%), full-time multifunctional farming in innovative mixed or organic production systems (~23%), and part-time farming where young farmers work off-farm and farm outside regular working hours (~24%). Using path analysis, we investigated which physical and psychological factors affect young farmers’ decisions to pursue these three farming types. The results show that non-monetary farming’s benefits are as important as monetary benefits. Education, farming and regular off-farm work experience, farm production, market and pest problems, and government support directly affect the farming types. These effects were also mediated by attitudes towards farming and net farming income. Young farmers choosing to pursue multifunctional farming have higher incomes, more often apply sophisticated technologies, and farm more sustainably than those choosing the other types of farming. This indicates that a shift from conventional rice production to more diversified production systems using innovative technologies is needed to sustain farming success and retain young people in the farming sector.

## Introduction

Socio-economic changes and technological advancements have led to a reduction in the number of young people involved in the farming sector across Europe, Asia, and America [1–5]. Young and highly educated people tend to take on well-paid jobs outside the farming sector [1, 3, 6]. In Western European countries, young farmers (under 40 years) account for between 4% (Portugal) and 22% (Austria) of the whole farm workforce [7]. In the USA, they account for ~6% [8], ~7% in Japan [9], and ~9% in South Korea [10]. A shift from smaller to larger farms has accompanied this decline in the young farming labour force due to increased production efficiency and economies of scale [11].

The decline of young people’s involvements in farming can be detrimental to farm productivity and food production [1–5, 12, 13]. Young farmers are more adaptable and often do better than older farmers in farming. This is because, firstly, young farmers are more risk tolerant and more likely to take out loans to invest in and expand their farm business than older farmers [1, 5]. Secondly, young farmers are willing to learn and therefore more likely to adopt innovative technologies, that help improve farm productivity and management [2, 4, 5, 13, 14]. Thirdly, young farmers are passionate about research and development, and the production and marketing of new, unique, diverse products, meeting high standards increasing their value using large collaborative networks [5, 14]. Lastly, young farmers are more likely to adhere to product and consumers health and safety, engaging in sustainable farming practices and conservation [2, 3, 5, 14].

Besides their advantageous farming behaviour for sustaining productivity, young farmers contribute to the liveability and well-being of rural communities by maintaining and disseminating site-specific farming knowledge and local culture [2, 3]. Young farmers are also needed to care for elderly parents and family-owned farmlands in a system where aged care and land inheritance remain a family responsibility [15, 16].

Against this background, our study aims to assess young farmers’ reasons for farming in the long-term, how they would like to farm, and what influences their decisions. More specifically, we aim to investigate the physical and psychological factors that influence their farming decisions. To meet these aims, we applied path analysis (PA) on data collected from interviews with young farmers in the Prachin Buri province of Thailand. The use of PA allowed us to reveal direct, indirect, and mediated factors affecting farming decisions. This information on why young farmers continue farming despite the general trend of farm abandonment can be used to formulate policies to encourage the long-term retention of young farmers in Thailand.

We used Thailand as a case-study because the number of farms is still high in emerging Asian countries, where the average age of farmers is increasing quite steeply [17]. Within ten years (2008 to 2018), the share of farmers under 40 years in Thailand decreased from ~16% to ~9% [18, 19]. Our study is novel in two ways. Firstly, it focuses on both physical and psychological factors [e.g., 3, 20, 21], while most studies focus on one or the other [1, 2, 4, 12, 22, 23]. Secondly, it is one of the few studies conducted in a developing country [3, 6, 21, 22]. While the problem of an ageing farming society is global, most studies on this subject so far were conducted in developed countries in Europe, the USA or Australia [1, 4, 12, 23]. The results of our study are therefore of relevance to policy-making in other Asian and developing countries that sooner or later may face a decline in interest of young people to farm, an exodus of youth from rural areas and ageing farmers left behind on the land.

## Conceptual framework

**Fig 1:**
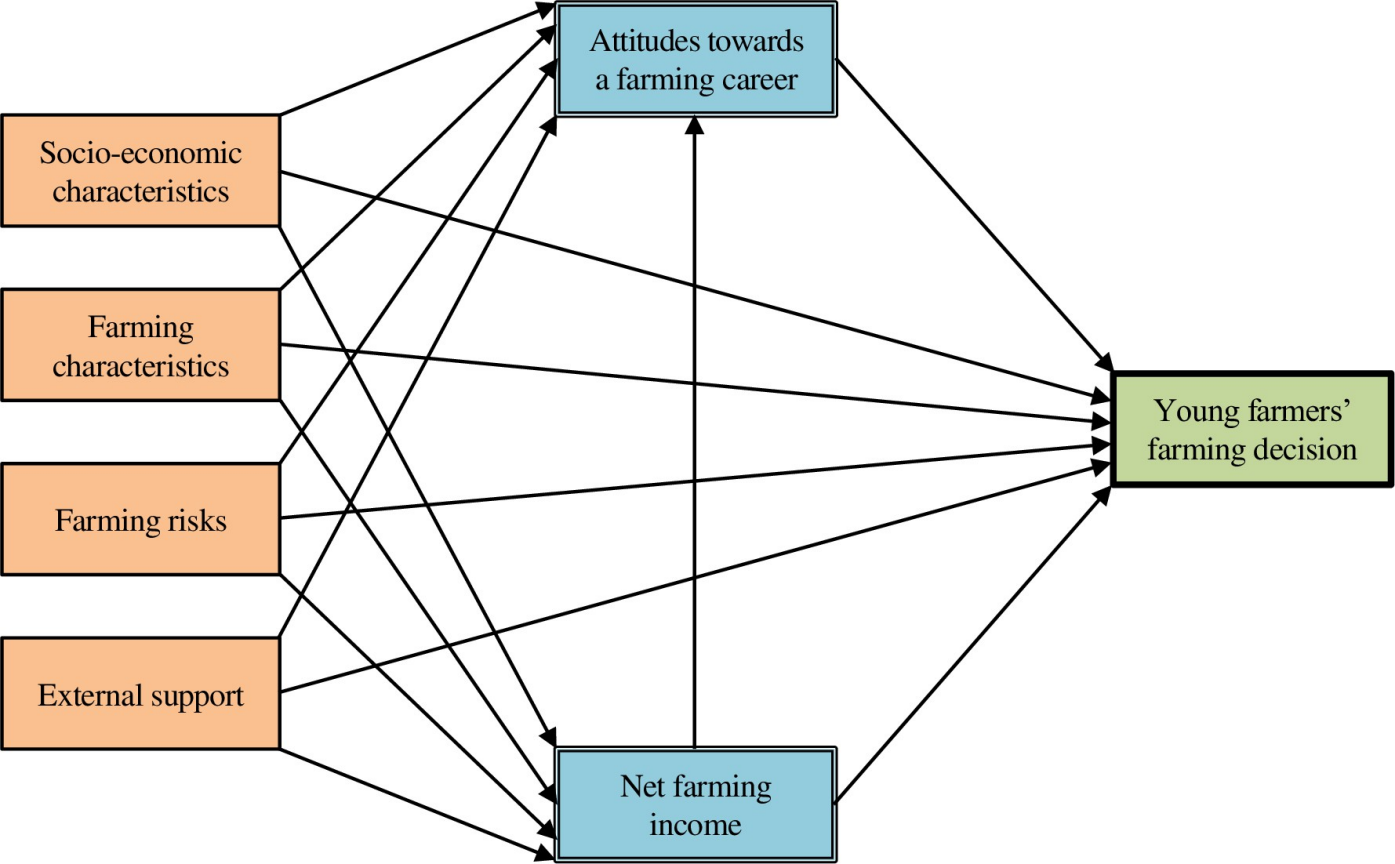
Path diagram of the hypotheses of the relationship between the factors and young farmers’ farming decision.

We constructed a conceptual framework that best explains young Thai farmers’ decisions to farm (Fig 1) by reviewing relevant literature on the determinants of 1) choice for an occupation, especially self-employment; 2) farm abandonment and entry; and 3) adoption of farming practices and innovations. These three aspects helped define the direct, indirect, and mediated factors that influenced young people’s decisions to continue farming. Although explaining people’s career decisions needed to incorporate as many relevant factors as possible, among these factors, income is one of the most important factors to explaining career decisions. Gross farming income (sales volume) was included as an explanatory variable in the analysis of Nadolnyak et al. [23], which showed gross income had a negative impact on young farmers’ decisions to exit farming. Farming income also varies with off-farm income, farm type (i.e., small-scale and subsistence farming, lifestyle farming), farm size and farming revenues, farm activity (e.g., livestock, cash crops, staple food), farm location, farm diversification, farmers’ age, farming experience, and government payments [24].

People’s behavioural decision-making, such as when pursuing an occupation or adopting a new farm production system, is a complex cognitive and learning process, depending on the interaction between people’s non-psychological and psychological factors [25]. Prior studies by Kang [20] and Parker [26] revealed that people’s attitudes towards risks and uncertainties of self-employment (e.g., farming) played a role in their decisions to become self-employed (e.g., farmers). Young people’s attitudes towards the farming sector, policy support (e.g., subsidy policy, farm entry policy, competitiveness policy, rural development policy), and farm entry barriers were correlated with their decisions to enter into and stay with farming [1, 20].

The close relation between farmers’ knowledge, perceptions, and attitudes about the benefits and risks of, the support and opposition by the important people surrounding them in, and their capabilities and constraints in farming innovations adoption influenced their decisions to adopt innovations. Such innovations include, for example, agroforestry, diversified farming, farming with multiple activities, and organic farming [25, 27–29].

Farmers’ attitudes towards being a farmer, land ownership, trust in the government, and farm expansion desires affect their decisions to participate in multifunctional farming, including on-farm product sale, nature conservation, agricultural production and trade services, and tourism [30]. Farmers’ attitudes were also affected by their personal and family characteristics, as well as their external environment characteristics, occupation and farming innovations characteristics, their social capital including farming organisations and network membership, communication and extension services [25, 30].

The literatures further showed that occupational decisions were directly influenced by the following factors:

- Individual factors–age, formal education, skills and abilities, gender, and race [12, 22, 23, 26, 28, 30],
- Family and household factors–marital status, number of dependent members, number of labour force among members, number of siblings, upbringing, and annual income [20, 22, 26, 28],
- Social factors–interactions with relatives, neighbours, and peers, as well as with occupational organisations and group memberships [26, 28–30],
- Farming and occupational factors–duration of farming, employment, or time in position, product type and diversity, family-owned business, expectation of getting a job, performance, farm and land size, relative product price to input price, off-farm employment and income, aims and strategies, and assets [12, 20, 22, 23, 26, 28–31],
- Location, natural, and environmental factors–distance to support agencies and urban areas, as well as economic and weather conditions [20, 22, 23, 29, 30], and
- Governmental and policy factors–non-financial and financial assistance [22, 23, 29, 30].

Our conceptual framework is showed in the path diagram in Fig 1. We grouped individual, family, and social factors into ‘socio-economic characteristics’ (Table 1) and hypothesise that these, as well as farming characteristics, farming risks and external support have a direct effect on the choice for a farming type. In addition, we hypothesis that attitudes towards farming and net farming income have a mediated effect on this choice.

**Table 1:** Definition and descriptive statistics of independent variables to be considered for inclusion in the models (n = 176).

Variable	Definition (coding)	Value
**Socio-economic characteristics**		
Gender	Being male (1 = yes, 0 = no) (%)	54
Age	Farmer’s age in years (mean; standard deviations)	40.4 (5.5)
Education	Completing education above Year 9 (1 = yes, 0 = no) (%)	62
Marriage	Being married (1 = yes, 0 = no) (%)	79
Children	Having dependent child (1 = yes, 0 = no) (%)	63
Experience	Own farming experience in years (mean; standard deviation)	11.6 (8.2)
Off-farm work	Regular off-farm work experience in years (mean; standard deviation)	8.9 (8.2)
Encouragement	Having ever been encouraged by parents to farm (1 = yes, 0 = no) (%)	12
**Farming characteristics**		
Production	Producing rice only (1 = yes, 0 = no) (%)	27
Size	Farmland size in rais (mean; standard deviation)	30.8 (40.3)
Tenure	Owning most of farmland (1 = yes, 0 = no) (%)	55
**Farming risks**		
Market	Facing falling product prices, rising costs, and insufficient funds (1 = yes, 0 = no) (%)	51
Pest	Facing plant disease, weed, insect, and animal pest outbreak (1 = yes, 0 = no) (%)	30
Climate	Facing irregular climate (1 = yes, 0 = no) (%)	39
Soil	Facing poor quality soil (1 = yes, 0 = no) (%)	6
**External support**		
Membership	Being occupational group member (1 = yes, 0 = no) (%)	89
Financial support	Receiving government financial support for farming (1 = yes, 0 = no) (%)	60
Non-financial support	Receiving knowledge and network support through YSF programme (1 = yes, 0 = no) (%)	35

## Methodology

### Ethics statement

Ethics approval was obtained from the Charles Darwin University Human Research Ethics Committee (H18028). Verbally informed consent was obtained from all respondents involved in this study. All data collected are de-identified.

### Study area

**Fig 2:**
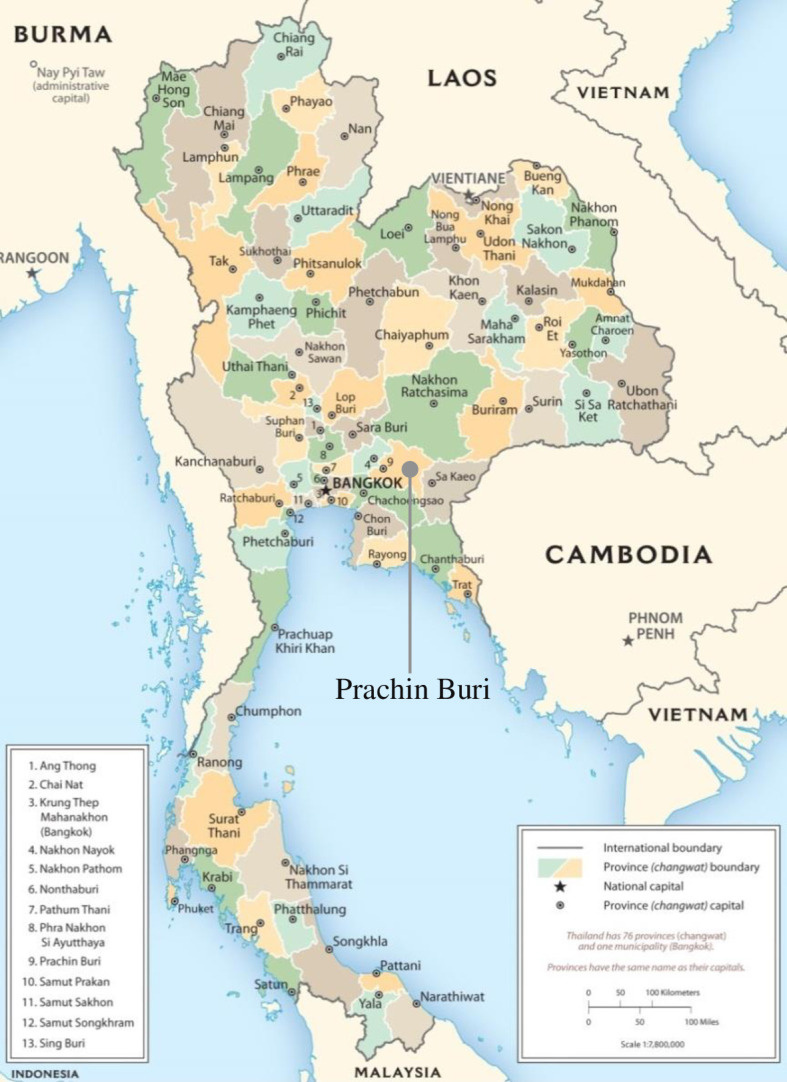
Study area in Prachin Buri province, Thailand. Note: Adapted from map produced by the U.S. Central Intelligence Agency; source: https://www.cia.gov/resources/map/thailand/.

The study was conducted in the Prachin Buri province in the central region of Thailand (Fig 2) [32]. We chose this province because of the relatively noticeable changes in its demographic and economic structure, as well as its role as one of the country’s breadbaskets. The population structure of the Prachin Buri province has shifted to a larger share of older people. In 1998, those aged 60 and older accounted for 9% of the province’s total population, while in 2019 they accounted for 17% [33]. The population is better educated than 18 years ago, with the proportion of employed people who have completed higher education (university degrees) increased from 9% in 2001 to 21% in 2019, and those who completed the compulsory education (Year 9) increased from 15% to 22% over the same period [34].

The Prachin Buri province is a regional important economic centre. In 2017, Prachin Buri had five large industrial areas with 252 factories, and another 713 factories scattered throughout the province [32]. The industrial and service sectors play a greater role in the province’s economy than the agricultural sector. Between 1990 and 2018, the province’s average annual growth rates of the industrial and service sectors were 16.5% and 5.3%, respectively, compared to -0.1% for the agricultural sector [35]. Between 2001 and 2019, the province’s average annual growth rate for people employed in the industrial and service sectors were 6.5% and 4.6%, respectively, compared to -2.3% in the agricultural sector [34]. During the same period, the province’s average annual unemployment rate was 1.2% [36]. These economic conditions indicate that young labourers in the province have increased opportunities to find employment in the non-agricultural sector [16]. This is one of the economic shifts the province has gone through since the 1990’s that make it an interesting case study.

However, the Prachin Buri province remains one of the more important agricultural production sites in the central region of Thailand. In 2018, more than half (54%) of the province’s total usable area is agricultural area, especially rice cultivation area (46%), making it the seventh largest of the 20 provinces in the region [37]. In 2016, more than 85% of farmers in the province adopted agricultural technologies, especially tractors and harvesting machines, to increase productivity [38]. In 2019, agricultural households in Prachin Buri had an average income of 66,881 baht per person per year (baht: monetary unit of Thailand; 1 USD = 33 baht) [39], which was much lower than the average personal income in the country (115,636 baht p.a.) [40] but above the country’s poverty line (33,155 baht per person per year) [41].

### Data collection

We purposively sampled 592 farmers for our entire research project on family farm succession planning of older farmers and farming aspirations of younger farmers. Of those, 544 responses were complete and valid, but for the analysis presented in this study, we used only a sub-sample of 176 responses from young farmers. As ‘young’ we defined farmers between the age of 18 and 45, in line with Thailand’s Ministry of Agriculture and Cooperatives recruitment strategy for the Young Smart Farmer (YSF) programme [42]. The lead author conducted face-to-face interviews with the selected farmers in 2018, using a semi-structured questionnaire.

### Data analysis

Path analysis (PA) was applied to analyse our data, based on the conceptual framework showed in the path diagram in Fig 1. PA is one of three core techniques of the structural equation modelling (SEM), the other two being the confirmatory factor analysis (CFA) and the evaluation of structural regression models (SR models) [43]. PA is capable of simultaneously estimating structural models’ parameters or testing series of hypotheses about the causal relationships between multiple exogenous (independent) and endogenous (mediation and dependent) variables observed together [43]. This technique was therefore suitable for our study because our model consisted only of structural models. In the application of this technique, we followed the SEM four stages that are involved in PA, as described by Kline [43], Gana and Broc [44], and Hair et al. [45].

The first stage was the specification of the structural models. This stage involved hypothesising about the dependence relationships between independent, mediation, and dependent variables. These hypotheses were depicted in the path diagram (Fig 1).

The independent variables to be considered for inclusion in our models (Fig 1: left) consisted of ratio-scaled variables: farmers’ age and farming and regular off-farm work experience (under socio-economic characteristics), and farm size (under farming characteristics; Table 1). The dichotomous-scaled variables included farmers’ educational attainment level and encouragement by parents to farm (under socio-economic characteristics), farm production (under farming characteristics), market problem (under farming risks), and receipt of government non-financial support through the YSF programme (under external support), among others.

**Fig 3:**
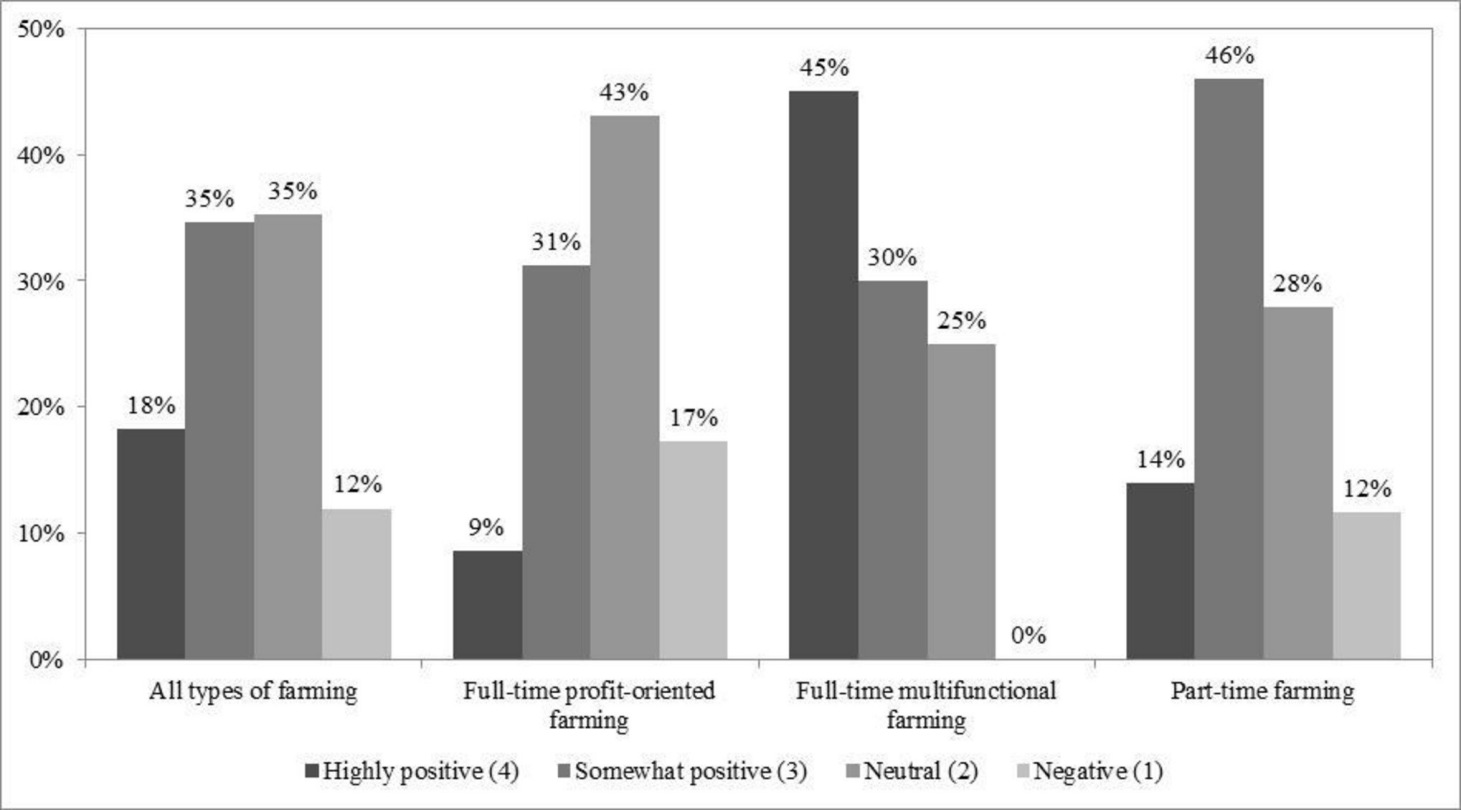
Attitudes towards farming. Note: 1) n = 176 and 2) Kruskal-Wallis H test (**χ**^**2**^) = 25.03, significant at 1% level.

**Fig 4:**
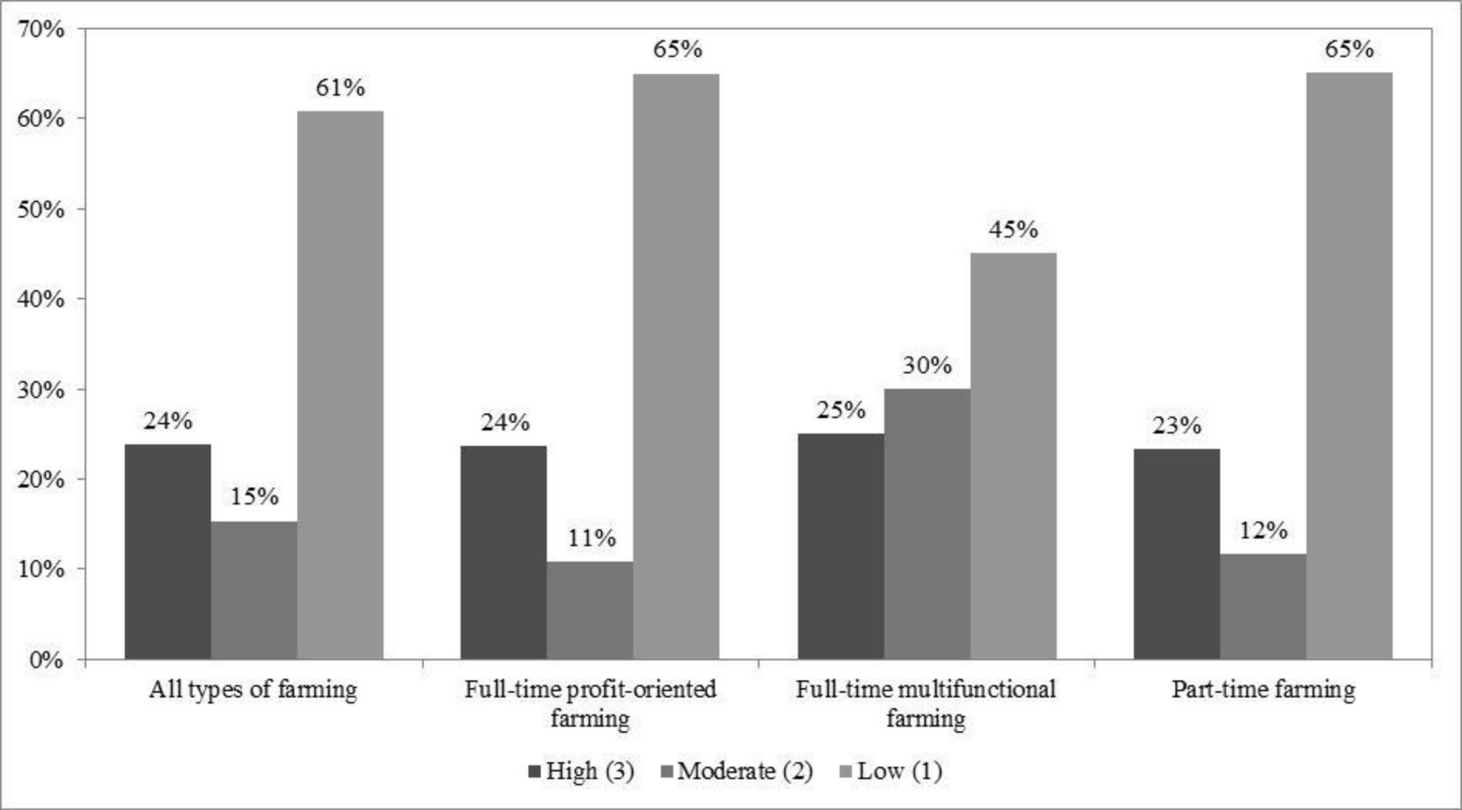
Net farming income per rai. Note: 1) n = 176 and 2) Kruskal-Wallis H test (**χ**^**2**^) = 3.17, insignificant at any level.

Variables used as mediators between independent and dependent variables (Fig 1: centre) included 1) farmers’ attitudes towards farming as a career and 2) their net farming income. Both of these variables were on an ordinal scale. We gauged attitudes by a number of non-monetary and monetary farming benefits. For this, farmers were asked *“What benefits do you think you receive from your farming career*?*”*. The different answers were counted and grouped into four attitude levels (1: negative [stating no benefit]; 2: neutral [stating one to two benefits]; 3: somewhat positive [stating three to four benefits]; 4: highly positive [stating five to eight benefits]; Fig 3). We expected that the more benefits farmers thought their farming careers provided them, the higher their attitude towards this occupation. To assess annual income, we used farmers’ net farming income, grouped into three levels (1: low [5,000 baht/rai or less]; 2: moderate [5,001–10,000 baht/rai]; 3: high [10,001 baht/rai or more]; rai: area measurement unit in Thailand; 1 km^2^ = 625 rai; Fig 4).

**Fig 5:**
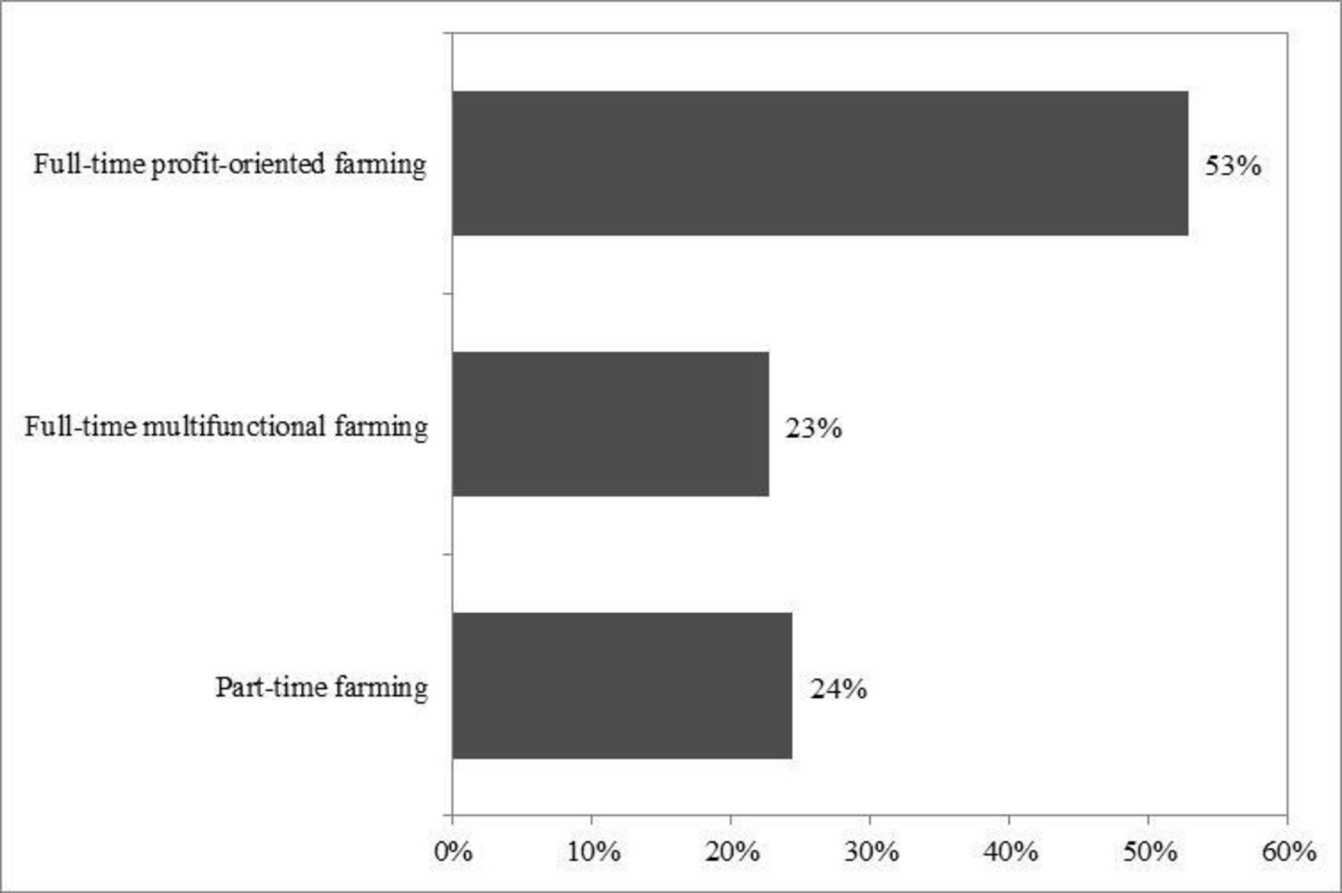
Types of farming (n = 176).

The dependent variable in the models (Fig 1: right) was the farming types on a dichotomous scale. Because we were interested in farmers’ decisions and reasons to continue farming, we asked as simple question, *“What is your main objective in continuing farming*?*”*, and allowed for open responses. We later categorised the open responses into three types of farming: 1) full-time profit-oriented farming, 2) full-time multifunctional farming, and 3) part-time farming (Fig 5). Each respondent fell into one of these categories.

The second stage was the estimation of the specified structural models, involving the estimation of the models’ parameters: path coefficients of independent variables. As our model’s mediation and dependent variables were ordinal and binary variables, we estimated the models by using generalised structural equation modelling (GSEM) on STATA, which by default uses maximum likelihood as the estimation method [46, 47]. Our models were separated into three models based on young farmers’ different farming types and estimated separately using the same data set.

The third stage was the assessment and selection of the estimated structural models with validity. At this stage, we computed and assessed the models’ a goodness-of-fit (GOF) index: second-order bias-corrected Akaike information criterion (AICc). A number of models with different independent variable components were estimated for each of three farming types, referred as the candidate models. The final model for each farming type was the candidate model with the lowest AICc, or the candidate model with less than three differences between its AICc and the lowest AICc of all models, as this indicated that the candidate model fitted the data comparatively well [48, 49].

The last stage was the interpretation of the final results of the structural models. In this last stage, we presented and discussed path diagrams with unstandardised coefficients and odds ratios and also explained the direct, indirect, and mediated effects between independent, mediation, and dependent variables.

## Results

### Sample description

Of 176 respondents, most (62%) completed education above Year 9 and received government financial support for their farming (60%; Table 1). Slightly more than half were man (54%), owned most of their farmland (55%), and faced market problems (51%). A minority had been encouraged by their parents to farm (12%), produced only rice (27%), faced pest problems (30%), and received training and networking support through the YSF programme (35%). On average, respondents had about 12 years of farming experience and nine years of regular off-farm work experience. Most respondents (53%) had positive attitudes towards farming (Fig 3). Nevertheless, their net farming income was low (Fig 4). None of the respondents stated an intention to exit farming in the near future.

### Farming decisions

Slightly more than half of the respondents (53%) were classified as full-time profit-oriented farmers with a focus on conventional rice production (Fig 5). About 23% also farmed full-time but with multifunctional purpose and in innovative mixed or organic production systems. Approximately 24% farmed part-time while having regular off-farm jobs, such as being a government official, a business owner, and a factory’s and private business employee. Multifunctional farmers were those whose primary farming objectives was not for the economic benefits, themselves, but for the non-economic benefits in their society. Their objectives included sharing products with neighbours, promoting good health by selling safe and organic products directly to consumers (e.g., running a stall at a weekly organic market or a showroom or a small restaurant attached to the farm), promoting sustainable farming practices by exchanging knowledge and experiences through different channels (e.g., learning centres), and providing recreational services (agritourism; S1 Table). They were also innovative, adopting sophisticated technologies, such as information and communication technologies (ICT; social media) to sell their products online, biological methods for dealing with plant diseases and pests, and environmentally-controlled houses for growing crops (S2 Table).

Full-time profit-oriented farmers had greater farming experience, were more likely to be solely rice producers, faced more market problems, and received more government financial support than those in the other two categories (S3 Table).

Those undertaking full-time and multifunctional farming had higher positive attitudes towards farming (Fig 3), earned higher incomes (Fig 4), were more likely to own most of their farmland, faced more pest problems, and were more likely to receive training and networking support through the YSF programme than the other respondents.

Those farming part-time were more likely to be man, better educated, influenced by their parents in their farming decisions and had more experience with regular off-farm work than respondents in the other two categories. Part-time farmers could have either profit-oriented or multifunctional farming objectives.

### Farming benefits

Farmers received both non-monetary and monetary benefits of their farming. The primary benefits common to most farmers across all three categories were that farming allowed them to work from their own homes (17%), made good use of their family-owned farmland and inherited their traditional family occupations (15%), economically supported themselves and their families (15%), and lived close to and took care of their dependent family members (14%; S4 Table). For each type of farmer, the most common benefit to the full-time multifunctional farmer was that farming allowed them to economically support themselves and their families (16%) while the most common benefit to full-time profit-oriented farmer and part-time farmer were that farming allowed them to work from their own homes (22% and 17%, respectively).

### Results of path analysis

#### Model fit and explanatory power

**Fig 6:**
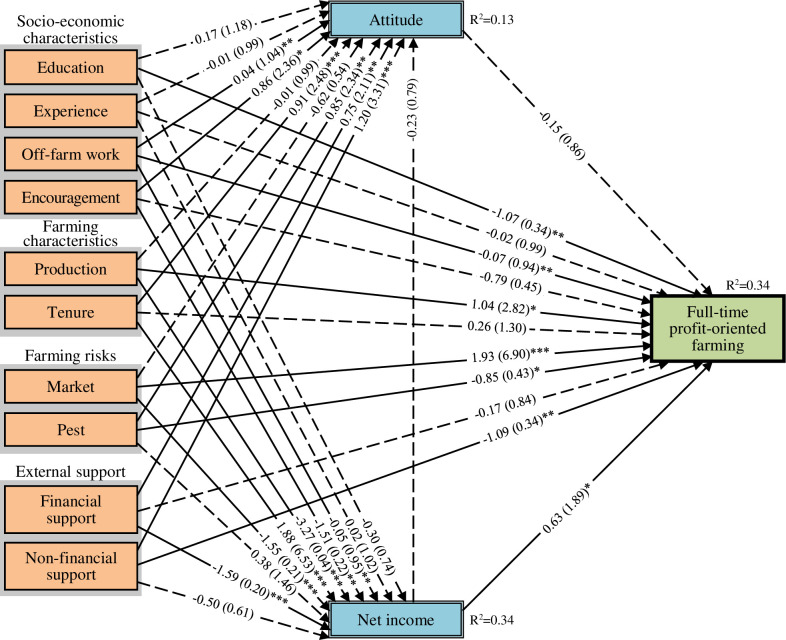
Path diagram of result of generalised structural equation modelling for full-time profit-oriented farming. Notes: 1) number of full-time profit-oriented farmers = 93, 2) number of samples (n) = 176, 3) *, **, *** significant at 10%, 5%, and 1% levels, 4) Dashed lines represent statistically insignificant path coefficients, 5) Numbers in the brackets were odds ratios, and 6) Second-order bias-corrected Akaike information criterion (AICc) = 876.71.

**Fig 7:**
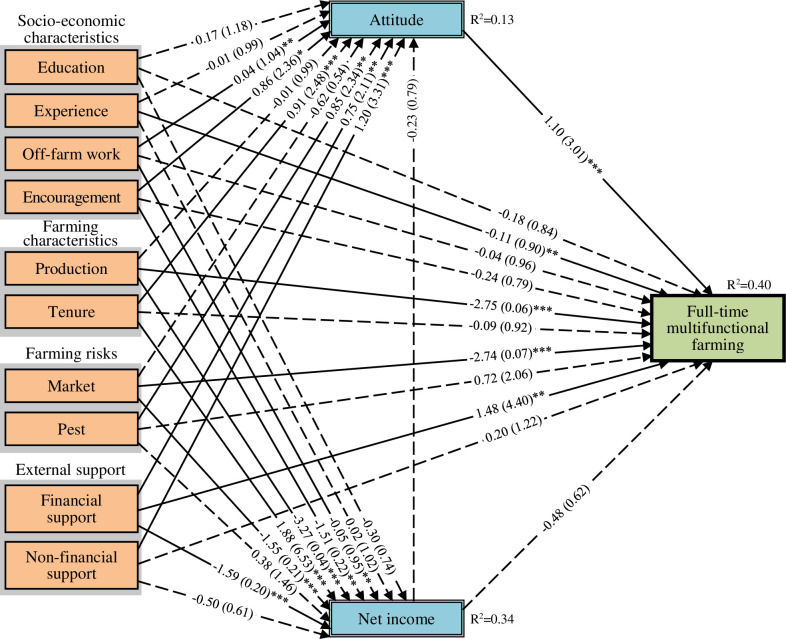
Path diagram of result of generalised structural equation modelling for full-time multifunctional farming. Notes: 1) number of full-time multifunctional farmers = 40, 2) number of samples (n) = 176, 3) *, **, *** significant at 10%, 5%, and 1% levels, 4) Dashed lines represent statistically insignificant path coefficients, 5) Numbers in the brackets were odds ratios, and 6) Second-order bias-corrected Akaike information criterion (AICc) = 830.97.

**Fig 8:**
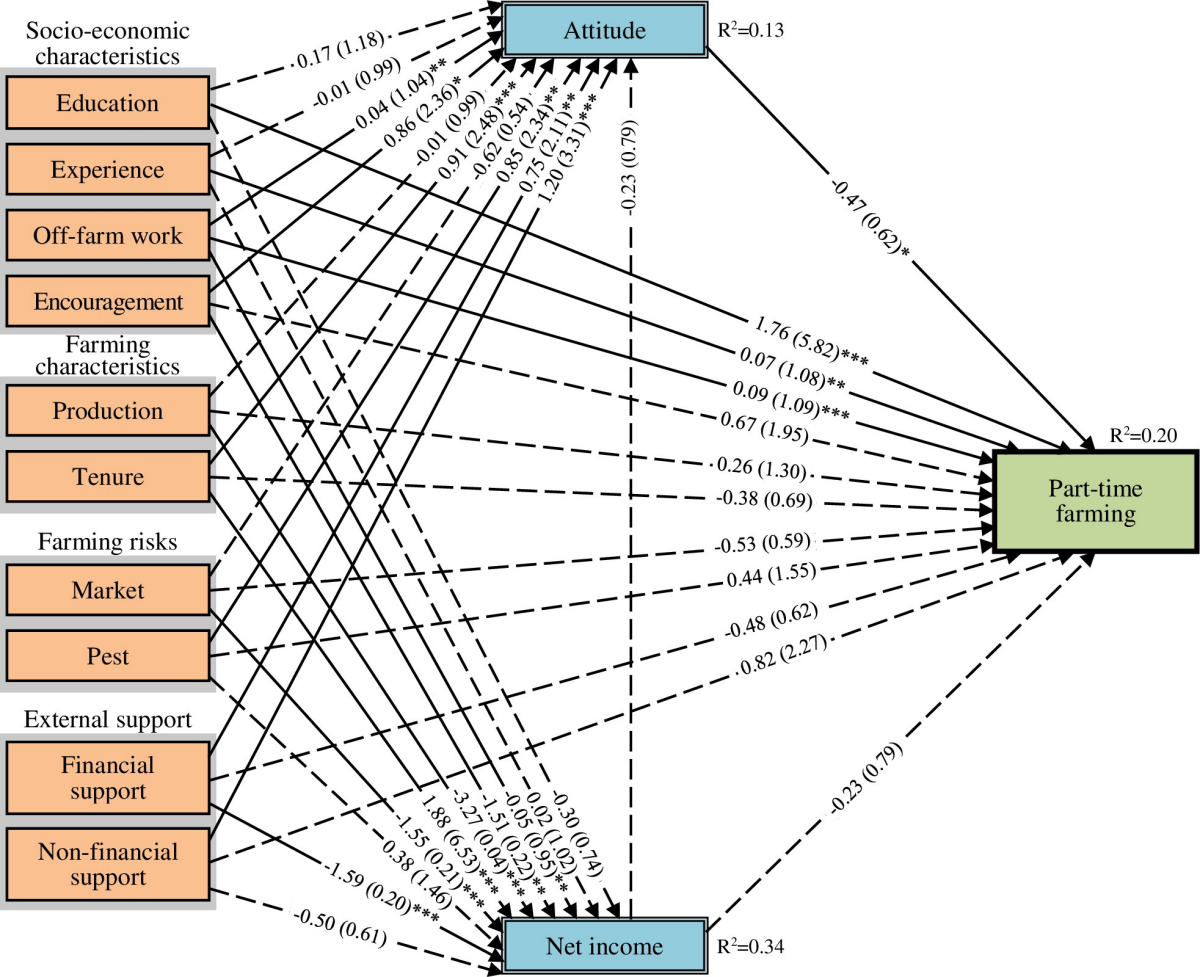
Path diagram of result of generalised structural equation modelling for part-time farming. Notes: 1) number of part-time farmers = 43, 2) number of samples (n) = 176, 3) *, **, *** significant at 10%, 5%, and 1% levels, 4) Dashed lines represent statistically insignificant path coefficients, 5) Numbers in the brackets were odds ratios, and 6) Second-order bias-corrected Akaike information criterion (AICc) = 873.86.

We included only statistically significant independent variables (S3 Table) into the models and selected the final models based on their AICc results. Our final models in the path diagrams in Figs 6–8 showed unstandardised coefficients and odds ratios (see S5 Table for the three final model results presented in tabular form with the variables’ standard errors). The final models fitted the data comparatively well, as their AICc results, when compared to the lowest AICc, were between 0.4 and 1.7, which were lower than a threshold of three (S6 Table) [48, 49]. The final models also did not violate the problem of interrelation of variables (Multicollinearity), as the variables’ values of variance inflation factor (VIF) were between 1.19 and 1.82, thus lower than a rule of thumb at ten.

All three final models had fair capacities to explain the variation in the mediation and dependent variables (Figs 6–8). Approximately 13% and 34% of the variation in farming attitudes and income, respectively, could be explained by the models. The models also explained about 34%, 40% and 20% of the variations in the decision to continue full-time profit-oriented farming, full-time multifunctional farming, and part-time farming, respectively.

#### Direct effects on farming decisions

We found that the full-time profit-oriented farming and full-time multifunctional farming types were directly affected by various factors related to socio-economic characteristics, farming characteristics, farming risks, and external support, while the part-time farming type was directly affected by only factors related to socio-economic characteristics (Figs 6–8). Education had inverse correlation with full-time profit-oriented farming but had direct correlation with part-time farming. Farmers with high levels of education were about six times more likely to choose part-time farming and to have regular off-farm work than those with low levels of education. Farming experience was negatively associated with full-time multifunctional farming while it was positively associated with part-time farming. Regular off-farm work experience negatively affected the choice for full-time profit-oriented farming while it positively affected the choice for part-time farming.

Farming production was positively associated with full-time profit-oriented farming but negatively associated with full-time multifunctional farming. Facing market problems was a positive predictor of full-time profit-oriented farming and a negative predictor of full-time innovative multifunctional farming. Farmers choosing full-time profit-oriented farming were about seven times more likely to face market problems than those in the other two categories. Pest problems had a negative influence on full-time profit-oriented farming.

Government financial support was positively associated with full-time innovative multifunctional farming, with these farmers being approximately four times more likely to receive government financial support than the other respondents. Government training and networking support through the YSF programme negatively influenced the choice for full-time profit-oriented farming.

#### Indirect effects on farming decisions

Attitudes towards farming as an occupation and net farming income mediated the effects of various factors related to socio-economic characteristics, farming characteristics, farming risks, and external support in all three models (Table 2). Through their higher net farming income, farmers who owned most of their land were approximately three times more likely than other farmers to continue full-time profit-oriented farming. Through their higher positive attitudes towards farming, farmers receiving government training and networking support through the YSF programme were approximately four times more likely than other farmers to pursue full-time multifunctional farming and 43% less likely than other farmers to pursue part-time farming.

**Table 2:** Result of Monte Carlo approach for computing and testing significance of indirect effects.

Indirect Effect	Full-time profit-oriented farming		Full-time multifunctional farming		Part-time farming	
	Mean Coef.	OR	Mean Coef.	OR	Mean Coef.	OR
**Net income as mediator**						
Off-farm work➝Net income➝Farming type	-0.03	0.97				
Encouragement➝Net income➝Farming type	-0.95*	0.39				
Production➝Net income➝ Farming type	-2.08*	0.13				
Tenure➝Net income➝ Farming type	1.19*	3.30				
Market➝Net income➝ Farming type	-0.98*	0.38				
Financial support➝Net income➝ Farming type	-1.01*	0.36				
**Attitude as mediator**						
Off-farm work➝Attitude➝ Farming type			0.05**	1.05	-0.02*	0.98
Encouragement➝Attitude➝ Farming type			0.95*	2.58	-0.41	0.67
Tenure➝Attitude➝ Farming type			1.00***	2.71	-0.43*	0.65
Pest➝Attitude➝ Farming type			0.94**	2.55	-0.40*	0.67
Financial support➝Attitude➝ Farming type			0.83**	2.28	-0.35	0.70
Non-financial support➝Attitude➝ Farming type			1.31***	3.72	-0.57*	0.57

Notes: 1) *, **, *** significant at 10%, 5%, and 1% level, 2) OR is odds ratio, and 3) Grey cells mean no indirect effect.

## Discussion

### Non-monetary benefits of farming

Farming is different from other occupations because it is more than an income-generating occupation; it is a way-of-life of people living in the countryside [50]. This was reflected in our results, which showed that farmers considered the non-monetary benefits of farming as important as the monetary benefits. In line with Milone and Ventura [14], Pinto-Correia et al. [50], Gennai-Schott et al. [51], Gillespie and Mishra [52], and Howley [53], we found that, overall, the primary benefits of farming included working from home and on family-owned farmland. This indicated a strong sense of place and belonging and a feeling of attachment to the farm, the location where farmers were born and where their families and intimate childhood friends live. Living in a rural community with good social and environmental conditions was also found to be a benefit in attracting and retaining farmers in other studies [i.e., 50, 51, 54].

Living close to family in order to be able to take care of ageing family members and children was another main non-monetary benefit of farming. This was expected within a Buddhist society’s norms, and many young farmers are likely to continue their parents’ farm due to these family commitments. This results in older people being looked after in the countryside, where aged care facilities are inadequate and widely inaccessible.

The other non-monetary farming benefits contributing to farmers’ continuous commitment included the good feeling of being their own boss, staying healthy by getting exercise through farming and working outdoors, and knowing that the food they consumed was their own produce and therefore safe to eat. This was underpinned by a positive attitude towards farming as a career by more than half of the farmers (53%), who think of themselves as doing a job they feel worth doing.

### Role of farming income

Monetary benefits are secondary to most farmers. Only those respondents who participated in full-time multifunctional farming saw the ability to economically support themselves and their families as the primary benefit of their farming career. On first sight, this is counter-intuitive, since it was the other full-time farming groups who were profit-oriented and for whom net farming income has a mediated impact on their farming practice. It is likely that most farmers within the full-time multifunctional group were not primarily cultivating rice (92%), the price of which is falling and that they were among the middle- and higher-income farmers (55%). They were also better educated, with many having relevant vocational certificates or university degrees, such as organic farming management, crop production technology, environmental science, agricultural science, or automotive mechanic. They have the skills to better plan and manage for long-term farming and make more money from farming in the future because of the knowledge and experience gained during their education than those who focused on short-term profits (labelled profit-oriented). At the same time, highly educated full-time multifunctional farmers embrace many non-income benefits (e.g., their own and consumers’ health, sharing products and knowledge with neighbours) [55] showing that revenues are not that important in their decisions to choose farming as occupation. Another possibility is that multifunctional farmers used loans from financial institutions (commercial bank, cooperative, and various funds within the community) to transform their farm into an innovate enterprise with a long-term vision and not only look at short-term profits needed to pay their bills. With these new sources of farm income, multifunctional farmers are ‘decoupled’ from market trends, such as volatile rice prices [56]. Receiving more government financial support also prevents multifunctional farmers from being directly affected by fluctuations of their net farming income.

### Full-time farmers

Three quarters of respondents farmed full-time. In line with our expectations expressed by the framework, education was directly correlated with the choice for the farming type. Profit-oriented farmers were the least educated and had the least regular off-farm experience. This is because they have been farming for a long time and decided to continue doing so, which was especially true for conventional rice farmers. Lacking both sufficient qualifications to get regular off-farm work and the knowledge readiness to adopt farming innovations explained why they did not adopt similar innovative methods to those adopted by full-time farmers in multifunctional systems.

Similar to Hennessy and Rehman [12], we also found that the practice by which farmers decided to farm depended on their product type. Farmers operating full-time profit-oriented farming were more likely to solely produce rice as their main source of income than those in the other two categories. Accustomed to rice farming due to their long experience, they were therefore reluctant to take the risks of starting over in another production systems [25, 29, 30, 57]. Hindrance from switching also arose from a lack of sufficient external support, farm location constraints, local limited market, high land adjustment cost, and labour shortages [58].

Full-time multifunctional farmers can be seen as an emerging group of young farmers in Thailand at present. Farmers in this category were relatively young and had less farming experience, with about 58% of those having five years or less farming experience. Many have returned recently to farming after either quitting regular off-farm work somewhere else due to family reasons (e.g., wanting to stay near family) and/or their regular off-farm works’ nature and environment reasons (e.g., unhealthy environment; nonautonomous, monotonous, and boring tasks; and unsupportive colleagues and supervisors). Multifunctional farming practice as an emerging concept can be farmers’ viable strategy to address the internal and external challenges and pressures on their farms, and to provide them with multiple income sources [59]. In addition to food production, these practices produced a wide range of other products and services to meet the needs of society, as described in Huttunen [15] and Wilson [55].

Multifunctional farming relies on a diversity of products, processes, and relations, which buffers weather and market related shocks [30, 59, 60]. Here, we found that full-time multifunctional farmers were less likely to solely produce rice than the other two categories of farmer. Their higher level of education allowed them to develop new ideas and adopt new innovations, thereby altering their farming styles from that of their parental generation. Their methods often include organic or sustainable farming, with ~80% of them using little or no chemicals (S2 Table). This gives them a greater chance of being economically successful in the long-term, thus enabling them to focus on other non-economic farming objectives, as found by Milone and Ventura [14].

While farming income source diversification explains why multifunctional farmers face fewer market problems than the other two categories, their innovative practices do not prevent them from the challenges of pests and diseases, to which multifunctional farmers were more susceptible to than profit-oriented framers focusing on rice monoculture, as the latter use more chemicals (S2 Table).

As expected, the likelihood of pursuing full-time farming, particularly multifunctional farming, increases with increasing positive attitudes towards farming. This is likely due to the following main reasons: 1) the opportunity for social networking through being members of the YSF network where farmers can share ideas, knowledge, experience and seek assistance, 2) increased awareness of the farming benefits, especially the non-monetary benefits, from farming encouragement by parents, 3) a strong feeling of belonging, sense of place and attachment to their own land, especially if the land has been owned by the family for many generations, and the wish to continue to utilise this land for food production, and 4) better health from pursuing low- or chemical-free farming. These, coupled with their reasons related to unpleasant experiences from prior regular off-farm work as described above, led them to take up farming and transform their farm into an innovative business, in line with their beliefs and the knowledge of achieving something worthwhile.

### Part-time farmers

About 24% of farmers were part-time farmers, whereby farming was mostly only the secondary occupation. These farmers may not wish to vacate their farmhouse, families, and farming, and therefore stay on the farm, farming outside of their regular working hours, such as in the early morning, late afternoon, and on weekends or public holidays. They are also attached to their home and land, maintaining their social networks and caring for their ageing parents. As such they still enjoy the benefits farming provides them but they are not dependant on the farming income and these benefits may therefore not be as high as in the case of the full-time multifunctional farmers.

As hypothesised in the framework, farmers’ decision to pursuing part-time farming were found to directly correlate with their education and farming and regular off-farm work experience [61]. Farmers in this category are the best educated, more so than the full-time multifunctional farmers, which is not surprising, as the good education gives them a greater chance of getting a well-paid regular off-farm job [30]. They have more experience with farming than those who do full-time multifunctional farming, which is likely because they grew up helping on the farms and continued farming to support their parents while receiving their education. Often, they also continue farming immediately after graduating from a college or university and start their first jobs in the non-agricultural sector while still living and working on their parents’ farm. They then decided to remain farming part-time to relief their parents from heavy farm work [62].

In Thailand, farm transfers occur mostly among all of the children of a household, with each child receiving some land, though each portion of inherited land is not large enough to sustain a full-time farmer. Well-educated young farmers follow a common trend, aiming to exit the agricultural sector altogether, e.g., after family commitments have been fulfilled. The increasing role of the non-agricultural sectors in the Thai economy due to economic and structural changes has led to an increasing demand for a general labour force and increasing wages, which are likely why part-time farmers never return to a solely farming lifestyle, as suggested by Deininger et al. [63].

Not surprisingly, the likelihood of pursuing part-time farming deceases with increasing positive attitudes towards farming. This is the opposite in the case of full-time multifunctional farming. If farmers in this category recognise that farming can provide them with the desired benefits compared to the benefits of non-farming, they are likely to return to full-time farming.

### Policy implications

An understanding of farmers’ aspirations is important when providing policies to support farmers in their objectives and to successfully secure regional food security and viable rural communities. Targeted support is needed for those who conventionally produce rice, an important stable food, and are vulnerable to market problems, particular price fluctuations, which lead to an unstable farming income. They are very profit oriented in the short-term, often without a long-term vision of adopting more innovative production systems and technologies (i.e., are trapped in their current situation). Policies should focus on providing direct finical support for long-term improvements, such as providing low interest or interest-free credits to delay the sale of paddy (raw product) when the price is falling; subsidies for rice production, harvest, and product quality improvement and management costs; and income protection insurance. A relatively high-priced specialty rice variety, such as low-sugar rice that is becoming of interest to health-conscious consumers and are suitable for diabetics, should be promoted to farmers to cultivate. Moreover, besides selling paddy to middlemen as many rice farmers do, they can be assisted in earning supplementary income by primary processing, thereby adding value to their products (e.g., rice packed in vacuum seal bags, rice flour, and rice noodle and paper) and directly selling to consumers. This can be done by supporting farmers to become members of the Farmers Institutes (e.g., community enterprise and cooperative), transferring knowledge on processing products and supporting related equipment and machinery to the Farmers Institutes, and publicising the processed products of the Farmers Institutes to the public.

Support is needed for profit-oriented farmers to alter their production systems from a conventional rice production system to a more sustainable production system, such as mixed or organic farming. This can be done by supporting them with, for example, knowledge on cultivating other crops and raising livestock, credit for land adjustment and purchase, facilitation of long-term land leases, other infrastructure (e.g., pond and greenhouse), inputs (e.g., seed or seedling), and market for distributing their products. This group of farmers can also benefit from policies and campaigns that increase their awareness of external support and funding opportunities, such as the YSF programme.

The future of farming, however, is likely to be multifunctional. Farmers pursuing full-time multifunctional farming are economically self-sufficient and had a more positive attitude towards farming, with a long-term vision of the sustainable and innovative production of a range of products, including rice. Possible policies to further support these farmers should be to develop their agricultural entrepreneurial skills with higher production efficiency and comprehensive business operations at the production, processing and distribution levels. The development of their own unique brand of their farming business can increase their competitiveness with other similar entrepreneurs in the market. Publicising their success stories to the public is another strategy to inspire potential young farmers become interested in farming, potentially through the training program and field trips delivered by the YSF programme. Their farms can also be developed into learning centres to provide opportunities for field trips and open farm days in which nearby conventional rice farmers can participate.

## Conclusion

Due to the socio-economic changes and technological advancements in many developing countries, a decline in the number of young farmers is taking place not only in the developed countries in Europe, the USA and Australia, but also in developing Asian countries, such as Thailand. This is likely to lead to food insecurity from a decline in farm productivity, and will have social consequences for the development of rural areas. Young people constitute a vital part of the liveability and economic growth of rural areas. Ensuring that young people who remain farmers are satisfied with their profession and are supported by the Thai government can counteract the general trend of rural youth out-migration.

This study applied path analysis to examine what physical and psychological factors affect young farmers’ decisions to continue farming and how they farm. We found that three-quarters of interviewed farmers continued full-time farming, albeit with different purposes. The profit-oriented farmers (53%) focused on rice production and on short-term income securing methods, possibly putting them at risk of instability in their economic situation. The other group of full-time farmers, the better educated and younger ones, based their decisions how to farm on their positive attitudes towards farming and the multiple non-monetary benefits. These farmers have made the multifunctional transitional processes of their farm business and maintain long-term plans, where farm income does not only depend on production, allowing new farming income opportunities to be explored and their economic situation to be relatively stable.

Multifunctional farming businesses provide many more benefits to the local society besides food production, such as knowledge transfer, awareness rising for safe and healthy food among the community’s members, agritourism and recreation, and environmental benefits from a more sustainable production. A quarter of farmers, meanwhile, had regular off-farm jobs and only farmed in their non-working hours. They felt a strong sense of place and wanted to continue to live on the farm, though without much farming engagement. These farmers were the most educated, with the most experience in both farming and off-farm work. Their farm succession and return to full-time farming remain on the crossroads, thus may be postponed, or may not happen at all. As different factors affect the different farming decisions of young Thai farmers, we conclude this study by recommending policies to support farmers’ financial viability and farming aspirations to keep current farmers in place, while attracting potential farmers to the industry. In other countries, especially developing countries with the same demographic, social, and economic structures, and which are facing the same problem of declining the number of young farmers as Thailand, researchers can apply this study’s methodology to conduct similar studies in these countries. Policy makers may also apply the policies recommended in this study to address the problems in their respective countries.

## Supporting information

S1 Table Classifying young farmers’ choices for farming by their farming objectives and time spent on farming (n = 176). (DOCX)

S2 Table Adoption of innovative farming methods and use of farming chemicals by farmers (n = 176). (DOCX)

S3 Table Characteristics of each type of farmers (n = 176). (DOCX)

S4 Table Perceived farming benefits. (DOCX)

S5 Table Result of the three final generalised structural equation models. (DOCX)

S6 Table Values of second-order bias-corrected Akaike information criterion (AICc) of the models for a) full-time profit-oriented farming type, b) full-time multifunctional farming type, and c) part-time farming type with different independent variable components.
(DOCX)
