# Supplementary material for: Multifunctional farming as successful pathway for the next generation of Thai farmers
Source: PLoS One. 2022 Apr 25;17(4):e0267351. doi: 10.1371/journal.pone.0267351 (PMC9037938; doi:10.1371/journal.pone.0267351)
Supplement: S1 Table — (DOCX) [file pone.0267351.s001.docx]

S1 Table. Classifying young farmers’ choices for farming by their farming objectives and time spent on farming (n=176).

| **Farming objective** | **Time spent on farming** | |
| --- | --- | --- |
|  | **Full-time** | **Part time** |
| **Profit-oriented objective** | | |
| - To get profit or at least not to make a loss, to have a business plan, to have low production costs, to get good/high/higher/quality yield, to distribute all/some products, to keep some products for use as seed, to be a direct distributor of products to consumers without having to go through middlemen, to cultivate and distribute better-tasting rice (*Leb Nok*) brought from Phatthalung province, to get good product prices, to get good/high/higher/better/predetermined income, to have a regular income for household use, to have some remaining income for saving, to get rid of debt, to be able to support oneself and family, to ensure family stability, to have better/stable career, to have own business, to be a modern farm adopting automatic systems and not relying on labours. | 52.8 | 13.6 |
| **Multifunctional objective** | | |
| - To get higher/quality yield; to keep some products for consumption and use as seed; to give away remainder of products to neighbours; to distribute remainder of products for income; to have enough food for personal and family consumption; to reduce family expenses and increase family income; to have income for own and family living; to have extra income for family expenses; to produce safe foods (less or no chemical use) for own, family, community, and general public consumption; to keep oneself, family members, and consumers healthy and free of cancer; to conduct organic farming, integrated farming, and agritourism; to promote organic farming; to be a learning centre for organic farming; to have a showroom for selling organic products to health lovers; to produce ingredients for family small restaurant; to lighten parents’ workload on farm and wait for taking over parents’ farm; to be own boss or not to be a subordinate; to have sustainable/successful career. | 22.7 | 10.8 |
