# Supplementary material for: Multifunctional farming as successful pathway for the next generation of Thai farmers
Source: PLoS One. 2022 Apr 25;17(4):e0267351. doi: 10.1371/journal.pone.0267351 (PMC9037938; doi:10.1371/journal.pone.0267351)
Supplement: S2 Table — (DOCX) [file pone.0267351.s002.docx]

S2 Table. Adoption of innovative farming methods and use of farming chemicals by farmers.

| **Items** | **All types of farming (n=176)** | **Full-time**  **profit-oriented farming**  **(n=93)** | **Full-time**  **multifunctional**  **farming**  **(n=40)** | **Part-time**  **farming**  **(n=43)** | **Test of difference**  **(**$\boldsymbol{\chi}^{\boldsymbol{2}}$**)** |
| --- | --- | --- | --- | --- | --- |
| Adopting innovative farming methods other than common machineries and chemicals (%) | 82.95 | 75.27 | 95.00 | 88.37 | 8.88** |
| Using no or less farming chemicals (%) | 53.41 | 38.71 | 80.00 | 60.47 | 23.30*** |

Note: 1) Innovative farming methods here refer to adopting agricultural machinery; agricultural chemicals; information and communication technologies (ICT); biological methods for improving soil and water quality, and dealing with plant diseases and pests; environmentally-controlled houses for growing crops, and raising livestock; management of farm irrigation systems; management of farmland for different usage purposes, and collection of farm statistical data for production planning; solar cells for generating electricity for farm use; hydroponics; and other more efficient cultivation and animal husbandry techniques, such as using mung bean peels to increase nitrogen in mushroom cultivation, and 2) *, **, *** significant at 10%, 5%, and 1% level.
