# Supplementary material for: Multifunctional farming as successful pathway for the next generation of Thai farmers
Source: PLoS One. 2022 Apr 25;17(4):e0267351. doi: 10.1371/journal.pone.0267351 (PMC9037938; doi:10.1371/journal.pone.0267351)
Supplement: S3 Table — (DOCX) [file pone.0267351.s003.docx]

S3 Table. Characteristics of each type of farmers.

| **Variable** | **Definition (coding)** | **Full-time**  **profit-oriented farming (n=93)** | | **Full-time**  **multifunctional**  **farming (n=40)** | | **Part-time**  **Farming (n=43)** | | **Test of difference** |
| --- | --- | --- | --- | --- | --- | --- | --- | --- |
|  |  | **Mean** | **SD** | **Mean** | **SD** | **Mean** | **SD** |  |
| **Socio-economic characteristics** | | | | | | | | |
| Gender | Being male (1=yes, 0=no) | 0.52 | 0.50 | 0.45 | 0.50 | 0.67 | 0.47 | 4.65* |
| Age | Farmer’s age (years) | 41.11 | 5.44 | 39.93 | 5.14 | 39.30 | 5.71 | 1.81 |
| Education | Completing education above Year 9 (1=yes, 0=no) | 0.43 | 0.50 | 0.80 | 0.41 | 0.86 | 0.35 | 30.27*** |
| Marriage | Being married (1=yes, 0=no) | 0.78 | 0.41 | 0.82 | 0.39 | 0.77 | 0.43 | 0.44 |
| Children | Having dependent child (1=yes, 0=no) | 0.60 | 0.49 | 0.65 | 0.48 | 0.67 | 0.47 | 0.74 |
| Experience | Own farming experience (years) | 13.62 | 8.48 | 7.48 | 6.75 | 11.09 | 7.27 | 8.72*** |
| Off-farm work | Regular off-farm work experience (years) | 6.97 | 8.26 | 9.63 | 8.11 | 12.21 | 6.98 | 6.67*** |
| Encouragement | Having ever been encouraged by parents to farm (1=yes, 0=no) | 0.08 | 0.27 | 0.13 | 0.34 | 0.21 | 0.41 | 5.04* |
| **Farming characteristics** | | | | | | | | |
| Production | Producing rice only (1=yes, 0=no) | 0.34 | 0.48 | 0.08 | 0.27 | 0.30 | 0.47 | 10.46*** |
| Size | Farmland size (rais) | 31.80 | 33.69 | 23.73 | 39.63 | 35.02 | 52.47 | 0.88 |
| Tenure | Owning most of farmland (1=yes, 0=no) | 0.48 | 0.50 | 0.70 | 0.46 | 0.56 | 0.50 | 5.29* |
| **Farming risks** | | | | | | | | |
| Market | Facing falling product prices, rising costs, and insufficient funds (1=yes, 0=no) | 0.74 | 0.44 | 0.10 | 0.30 | 0.37 | 0.49 | 50.17*** |
| Pest | Facing plant disease, weed, insect, and animal pest outbreak (1=yes, 0=no) | 0.19 | 0.40 | 0.48 | 0.51 | 0.35 | 0.48 | 11.42*** |
| Climate | Facing irregular climate (1=yes, 0=no) | 0.34 | 0.48 | 0.38 | 0.49 | 0.51 | 0.51 | 3.53 |
| Soil | Facing poor quality soil (1=yes, 0=no) | 0.05 | 0.23 | 0.05 | 0.22 | 0.09 | 0.29 | 0.91 |
| **External support** | | | | | | | | |
| Membership | Being occupational group member (1=yes, 0=no) | 0.89 | 0.31 | 0.92 | 0.27 | 0.84 | 0.37 | 1.66 |
| Financial support | Receiving government financial support for farming (1=yes, 0=no) | 0.68 | 0.47 | 0.55 | 0.50 | 0.47 | 0.51 | 5.97* |
| Non-financial support | Receiving knowledge and network support through YSF programme (1=yes, 0=no) | 0.17 | 0.38 | 0.58 | 0.50 | 0.51 | 0.51 | 26.90*** |

Notes: 1) Chi-square test and One-Way ANOVA were applied to test the difference of nominal and ratio variables, respectively, among the farmer groups, and 2) *, **, *** significant at 10%, 5%, and 1% level.
