# Supplementary material for: Multifunctional farming as successful pathway for the next generation of Thai farmers
Source: PLoS One. 2022 Apr 25;17(4):e0267351. doi: 10.1371/journal.pone.0267351 (PMC9037938; doi:10.1371/journal.pone.0267351)
Supplement: S4 Table — (DOCX) [file pone.0267351.s004.docx]

S4 Table. Perceived farming benefits.

| **Benefit** | **All types of farming^1^** | **Full-time**  **profit-oriented farming^2^** | **Full-time multifunctional farming^3^** | **Part-time farming ^4^** |
| --- | --- | --- | --- | --- |
| 1. Working from their own homes. | **16.47** | **22.33** | 7.11 | **16.97** |
| 1. Making good use of their family-owned farmland and inheriting their traditional family occupations. | **15.11** | **15.33** | **14.72** | **15.15** |
| 1. Economically supporting themselves and their families. | **14.95** | **15.00** | **16.24** | **13.33** |
| 1. Living close to and taking care of their family members. | **13.60** | **17.00** | 9.64 | **12.12** |
| 1. Pursuing independent and non-monotonous career and becoming their own bosses. | 10.57 | 13.00 | 10.15 | 6.67 |
| 1. Contributing to keeping farmers themselves and their customers healthy. | 8.61 | 3.67 | **15.23** | 9.70 |
| 1. Being source of food for farmers’ household consumption. | 6.95 | 2.33 | **12.69** | 8.48 |
| 1. Living in rural communities with generous and supportive neighbours and relatively low daily living expense. | 6.04 | 4.67 | 6.60 | 7.88 |
| 1. Living close to the nature and in a good environment. | 4.83 | 4.00 | 4.06 | 7.27 |
| 1. Being no longer hard work as there are many available contractors and labour-saving machines today. | 2.87 | 2.67 | 3.55 | 2.42 |

Notes: 1) ^1,2,3,4^ n=662, 300, 197, and 165, respectively, as farmers chose more than one answer, and 2) Bold numbers are the top four largest percentages.
