# Supplementary material for: Multifunctional farming as successful pathway for the next generation of Thai farmers
Source: PLoS One. 2022 Apr 25;17(4):e0267351. doi: 10.1371/journal.pone.0267351 (PMC9037938; doi:10.1371/journal.pone.0267351)
Supplement: S5 Table — (DOCX) [file pone.0267351.s005.docx]

S5 Table. Result of the three final generalised structural equation models.

| **Variable** | **Net income** | | | **Attitude** | | | **Full-time**  **profit-oriented**  **farming** | | | **Full-time**  **multifunctional**  **farming** | | | **Part-time**  **farming** | | |
| --- | --- | --- | --- | --- | --- | --- | --- | --- | --- | --- | --- | --- | --- | --- | --- |
|  | **Coef.** | **SE** | **OR** | **Coef.** | **SE** | **OR** | **Coef.** | **SE** | **OR** | **Coef.** | **SE** | **OR** | **Coef.** | **SE** | **OR** |
| **Mediation variable** | | | | | | | | | | | | | | | |
| Attitude |  |  |  |  |  |  | -0.15 | 0.25 | 0.86 | 1.10*** | 0.36 | 3.01 | -0.47* | 0.26 | 0.62 |
| Net income |  |  |  | -0.23 | 0.23 | 0.79 | 0.63* | 0.33 | 1.89 | -0.48 | 0.34 | 0.62 | -0.23 | 0.31 | 0.79 |
| **Independent variable** | | | | | | | | | | | | | | | |
| Education | -0.30 | 0.48 | 0.74 | 0.17 | 0.36 | 1.18 | -1.07** | 0.47 | 0.34 | -0.18 | 0.65 | 0.84 | 1.76*** | 0.59 | 5.82 |
| Experience | 0.02 | 0.03 | 1.02 | -0.01 | 0.02 | 0.99 | -0.02 | 0.03 | 0.99 | -0.11** | 0.04 | 0.90 | 0.07** | 0.03 | 1.08 |
| Off-farm work | -0.05** | 0.03 | 0.95 | 0.04** | 0.02 | 1.04 | -0.07** | 0.03 | 0.94 | -0.04 | 0.03 | 0.96 | 0.09*** | 0.03 | 1.09 |
| Encouragement | -1.51** | 0.72 | 0.22 | 0.86* | 0.49 | 2.36 | -0.79 | 0.68 | 0.45 | -0.24 | 0.81 | 0.79 | 0.67 | 0.62 | 1.95 |
| Production | -3.27*** | 1.07 | 0.04 | -0.01 | 0.37 | 0.99 | 1.04* | 0.56 | 2.82 | -2.75*** | 0.88 | 0.06 | 0.26 | 0.53 | 1.30 |
| Tenure | 1.88*** | 0.47 | 6.53 | 0.91*** | 0.34 | 2.48 | 0.26 | 0.47 | 1.30 | -0.09 | 0.61 | 0.92 | -0.38 | 0.48 | 0.69 |
| Market | -1.55*** | 0.46 | 0.21 | -0.62 | 0.38 | 0.54 | 1.93*** | 0.50 | 6.90 | -2.74*** | 0.74 | 0.07 | -0.53 | 0.52 | 0.59 |
| Pest | 0.38 | 0.41 | 1.46 | 0.85** | 0.35 | 2.34 | -0.85* | 0.46 | 0.43 | 0.72 | 0.50 | 2.06 | 0.44 | 0.48 | 1.55 |
| Financial support | -1.59*** | 0.44 | 0.20 | 0.75** | 0.37 | 2.11 | -0.17 | 0.50 | 0.84 | 1.48** | 0.61 | 4.40 | -0.48 | 0.47 | 0.62 |
| Non-financial support | -0.50 | 0.48 | 0.61 | 1.20*** | 0.39 | 3.31 | -1.09** | 0.52 | 0.34 | 0.20 | 0.58 | 1.22 | 0.82 | 0.53 | 2.27 |

Notes: 1) *, **, *** significant at 10%, 5%, and 1% level, 2) SE is standard errors, 3) OR is odds ratio, and 4) Grey cells mean no relationship hypothesis between variables.
