# Supplementary material for: Multifunctional farming as successful pathway for the next generation of Thai farmers
Source: PLoS One. 2022 Apr 25;17(4):e0267351. doi: 10.1371/journal.pone.0267351 (PMC9037938; doi:10.1371/journal.pone.0267351)
Supplement: S6 Table — Values of second-order bias-corrected Akaike information criterion (AICc) of the models for a) full-time profit-oriented farming type, b) full-time multifunctional farming type, and c) part-time farming type with different independent variable components. (DOCX) [file pone.0267351.s006.docx]

**S6 Table. Values of second-order bias-corrected Akaike information criterion (AICc) of the models for a) full-time profit-oriented farming type, b) full-time multifunctional farming type, and c) part-time farming type with different independent variable components.**

| **Variable** | **CM1** | **CM2** | **CM3** | **CM4** | **CM5** | **CM6** | **CM7** | **CM8** | **CM9** | **CM10** | **CM11** | **FM** | **CM12** | **CM13** | **CM14** | **CM15** |
| --- | --- | --- | --- | --- | --- | --- | --- | --- | --- | --- | --- | --- | --- | --- | --- | --- |
|  | **a) Full-time profit-oriented farming type** | | | | | | | | | | | | | | | |
| **Mediation variable** | | | | | | | | | | | | | | | | |
| Attitude | 🗸 | 🗸 | 🗸 | 🗸 | 🗸 | 🗸 | 🗸 | 🗸 | 🗸 | 🗸 | 🗸 | 🗸 | 🗸 | 🗸 | 🗸 | 🗸 |
| Net income | 🗸 | 🗸 | 🗸 | 🗸 | 🗸 | 🗸 | 🗸 | 🗸 | 🗸 | 🗸 | 🗸 | 🗸 | 🗸 | 🗸 | 🗸 | 🗸 |
| **Independent variable** | | | | | | | | | | | | | | | | |
| Gender |  | 🗸 |  |  |  |  |  |  | 🗸 | 🗸 | 🗸 |  | 🗸 | 🗸 | 🗸 | 🗸 |
| Education | 🗸 | 🗸 | 🗸 | 🗸 | 🗸 | 🗸 | 🗸 | 🗸 | 🗸 | 🗸 | 🗸 | 🗸 | 🗸 | 🗸 | 🗸 | 🗸 |
| Experience | 🗸 | 🗸 | 🗸 | 🗸 | 🗸 | 🗸 | 🗸 | 🗸 | 🗸 | 🗸 | 🗸 | 🗸 | 🗸 | 🗸 | 🗸 | 🗸 |
| Off-farm work | 🗸 | 🗸 | 🗸 | 🗸 | 🗸 | 🗸 | 🗸 | 🗸 | 🗸 | 🗸 | 🗸 | 🗸 | 🗸 | 🗸 | 🗸 | 🗸 |
| Encouragement |  |  | 🗸 |  |  |  | 🗸 | 🗸 |  |  | 🗸 | 🗸 |  | 🗸 | 🗸 | 🗸 |
| Production | 🗸 | 🗸 | 🗸 | 🗸 | 🗸 | 🗸 | 🗸 | 🗸 | 🗸 | 🗸 | 🗸 | 🗸 | 🗸 | 🗸 | 🗸 | 🗸 |
| Tenure |  |  |  | 🗸 |  | 🗸 |  | 🗸 |  | 🗸 |  | 🗸 | 🗸 |  | 🗸 | 🗸 |
| Market | 🗸 | 🗸 | 🗸 | 🗸 | 🗸 | 🗸 | 🗸 | 🗸 | 🗸 | 🗸 | 🗸 | 🗸 | 🗸 | 🗸 | 🗸 | 🗸 |
| Pest | 🗸 | 🗸 | 🗸 | 🗸 | 🗸 | 🗸 | 🗸 | 🗸 | 🗸 | 🗸 | 🗸 | 🗸 | 🗸 | 🗸 | 🗸 | 🗸 |
| Financial support |  |  |  |  | 🗸 | 🗸 | 🗸 |  | 🗸 |  |  | 🗸 | 🗸 | 🗸 |  | 🗸 |
| Non-financial support | 🗸 | 🗸 | 🗸 | 🗸 | 🗸 | 🗸 | 🗸 | 🗸 | 🗸 | 🗸 | 🗸 | 🗸 | 🗸 | 🗸 | 🗸 | 🗸 |
| **Independent variable number** | 7 | 8 | 8 | 8 | 8 | 9 | 9 | 9 | 9 | 9 | 9 | 10 | 10 | 10 | 10 | 11 |
| **AIC** | 888.26 | 891.11 | 886.99 | 867.16 | 876.47 | 857.19 | 873.17 | 865.95 | 880.47 | 869.50 | 889.94 | 853.77 | 860.91 | 877.17 | 868.27 | 857.43 |
| **AICc** | 901.09 | 906.92 | 902.79 | 882.96 | 892.27 | 876.35 | 892.34 | 885.12 | 899.63 | 888.67 | 909.10 | 876.71 | 883.85 | 900.11 | 891.21 | 884.59 |
| **AICc_CMi_-AICc_CM6_** | 24.74 | 30.57 | 26.44 | 6.61 | 15.92 | 0.00 | 15.99 | 8.77 | 23.28 | 12.32 | 32.75 | 0.36 | 7.50 | 23.76 | 14.86 | 8.24 |

Note: 1) CM: candidate model; FM: final model; AIC: Akaike information criterion; AICc: second-order bias-corrected AIC, and 2) Grey cells mean variables are not included in the model.

| **Variable** | **CM1** | **CM2** | **CM3** | **CM4** | **CM5** | **CM6** | **CM7** | **CM8** | **CM9** | **CM10** | **CM11** | **FM** | **CM12** | **CM13** | **CM14** | **CM15** |
| --- | --- | --- | --- | --- | --- | --- | --- | --- | --- | --- | --- | --- | --- | --- | --- | --- |
|  | **b) Full-time multifunctional farming type** | | | | | | | | | | | | | | | |
| **Mediation variable** | | | | | | | | | | | | | | | | |
| Attitude | 🗸 | 🗸 | 🗸 | 🗸 | 🗸 | 🗸 | 🗸 | 🗸 | 🗸 | 🗸 | 🗸 | 🗸 | 🗸 | 🗸 | 🗸 | 🗸 |
| Net income | 🗸 | 🗸 | 🗸 | 🗸 | 🗸 | 🗸 | 🗸 | 🗸 | 🗸 | 🗸 | 🗸 | 🗸 | 🗸 | 🗸 | 🗸 | 🗸 |
| **Independent variable** | | | | | | | | | | | | | | | | |
| Gender |  | 🗸 |  |  |  |  |  |  | 🗸 | 🗸 | 🗸 |  | 🗸 | 🗸 | 🗸 | 🗸 |
| Education | 🗸 | 🗸 | 🗸 | 🗸 | 🗸 | 🗸 | 🗸 | 🗸 | 🗸 | 🗸 | 🗸 | 🗸 | 🗸 | 🗸 | 🗸 | 🗸 |
| Experience | 🗸 | 🗸 | 🗸 | 🗸 | 🗸 | 🗸 | 🗸 | 🗸 | 🗸 | 🗸 | 🗸 | 🗸 | 🗸 | 🗸 | 🗸 | 🗸 |
| Off-farm work | 🗸 | 🗸 | 🗸 | 🗸 | 🗸 | 🗸 | 🗸 | 🗸 | 🗸 | 🗸 | 🗸 | 🗸 | 🗸 | 🗸 | 🗸 | 🗸 |
| Encouragement |  |  | 🗸 |  |  |  | 🗸 | 🗸 |  |  | 🗸 | 🗸 |  | 🗸 | 🗸 | 🗸 |
| Production | 🗸 | 🗸 | 🗸 | 🗸 | 🗸 | 🗸 | 🗸 | 🗸 | 🗸 | 🗸 | 🗸 | 🗸 | 🗸 | 🗸 | 🗸 | 🗸 |
| Tenure |  |  |  | 🗸 |  | 🗸 |  | 🗸 |  | 🗸 |  | 🗸 | 🗸 |  | 🗸 | 🗸 |
| Market | 🗸 | 🗸 | 🗸 | 🗸 | 🗸 | 🗸 | 🗸 | 🗸 | 🗸 | 🗸 | 🗸 | 🗸 | 🗸 | 🗸 | 🗸 | 🗸 |
| Pest | 🗸 | 🗸 | 🗸 | 🗸 | 🗸 | 🗸 | 🗸 | 🗸 | 🗸 | 🗸 | 🗸 | 🗸 | 🗸 | 🗸 | 🗸 | 🗸 |
| Financial support |  |  |  |  | 🗸 | 🗸 | 🗸 |  | 🗸 |  |  | 🗸 | 🗸 | 🗸 |  | 🗸 |
| Non-financial support | 🗸 | 🗸 | 🗸 | 🗸 | 🗸 | 🗸 | 🗸 | 🗸 | 🗸 | 🗸 | 🗸 | 🗸 | 🗸 | 🗸 | 🗸 | 🗸 |
| **Independent variable number** | 7 | 8 | 8 | 8 | 8 | 9 | 9 | 9 | 9 | 9 | 9 | 10 | 10 | 10 | 10 | 11 |
| **AIC** | 847.70 | 850.71 | 847.40 | 826.62 | 829.30 | 810.15 | 827.14 | 826.59 | 834.65 | 829.00 | 850.48 | 808.03 | 815.16 | 832.53 | 828.89 | 813.02 |
| **AICc** | 860.53 | 866.51 | 863.21 | 842.43 | 845.10 | 829.31 | 846.30 | 845.75 | 853.81 | 848.17 | 869.64 | 830.97 | 838.10 | 855.48 | 851.83 | 840.18 |
| **AICc_CMi_-AICc_CM6_** | 31.22 | 37.2 | 33.9 | 13.12 | 15.79 | 0.00 | 16.99 | 16.44 | 24.5 | 18.86 | 40.33 | 1.66 | 8.79 | 26.17 | 22.52 | 10.87 |

Note: 1) CM: candidate model; FM: final model; AIC: Akaike information criterion; AICc: second-order bias-corrected AIC, and 2) Grey cells mean variables are not included in the model.

| **Variable** | **CM1** | **CM2** | **CM3** | **CM4** | **CM5** | **CM6** | **CM7** | **CM8** | **CM9** | **CM10** | **CM11** | **FM** | **CM12** | **CM13** | **CM14** | **CM15** |
| --- | --- | --- | --- | --- | --- | --- | --- | --- | --- | --- | --- | --- | --- | --- | --- | --- |
|  | **c) Part-time farming type** | | | | | | | | | | | | | | | |
| **Mediation variable** | | | | | | | | | | | | | | | | |
| Attitude | 🗸 | 🗸 | 🗸 | 🗸 | 🗸 | 🗸 | 🗸 | 🗸 | 🗸 | 🗸 | 🗸 | 🗸 | 🗸 | 🗸 | 🗸 | 🗸 |
| Net income | 🗸 | 🗸 | 🗸 | 🗸 | 🗸 | 🗸 | 🗸 | 🗸 | 🗸 | 🗸 | 🗸 | 🗸 | 🗸 | 🗸 | 🗸 | 🗸 |
| **Independent variable** | | | | | | | | | | | | | | | | |
| Gender |  | 🗸 |  |  |  |  |  |  | 🗸 | 🗸 | 🗸 |  | 🗸 | 🗸 | 🗸 | 🗸 |
| Education | 🗸 | 🗸 | 🗸 | 🗸 | 🗸 | 🗸 | 🗸 | 🗸 | 🗸 | 🗸 | 🗸 | 🗸 | 🗸 | 🗸 | 🗸 | 🗸 |
| Experience | 🗸 | 🗸 | 🗸 | 🗸 | 🗸 | 🗸 | 🗸 | 🗸 | 🗸 | 🗸 | 🗸 | 🗸 | 🗸 | 🗸 | 🗸 | 🗸 |
| Off-farm work | 🗸 | 🗸 | 🗸 | 🗸 | 🗸 | 🗸 | 🗸 | 🗸 | 🗸 | 🗸 | 🗸 | 🗸 | 🗸 | 🗸 | 🗸 | 🗸 |
| Encouragement |  |  | 🗸 |  |  |  | 🗸 | 🗸 |  |  | 🗸 | 🗸 |  | 🗸 | 🗸 | 🗸 |
| Production | 🗸 | 🗸 | 🗸 | 🗸 | 🗸 | 🗸 | 🗸 | 🗸 | 🗸 | 🗸 | 🗸 | 🗸 | 🗸 | 🗸 | 🗸 | 🗸 |
| Tenure |  |  |  | 🗸 |  | 🗸 |  | 🗸 |  | 🗸 |  | 🗸 | 🗸 |  | 🗸 | 🗸 |
| Market | 🗸 | 🗸 | 🗸 | 🗸 | 🗸 | 🗸 | 🗸 | 🗸 | 🗸 | 🗸 | 🗸 | 🗸 | 🗸 | 🗸 | 🗸 | 🗸 |
| Pest | 🗸 | 🗸 | 🗸 | 🗸 | 🗸 | 🗸 | 🗸 | 🗸 | 🗸 | 🗸 | 🗸 | 🗸 | 🗸 | 🗸 | 🗸 | 🗸 |
| Financial support |  |  |  |  | 🗸 | 🗸 | 🗸 |  | 🗸 |  |  | 🗸 | 🗸 | 🗸 |  | 🗸 |
| Non-financial support | 🗸 | 🗸 | 🗸 | 🗸 | 🗸 | 🗸 | 🗸 | 🗸 | 🗸 | 🗸 | 🗸 | 🗸 | 🗸 | 🗸 | 🗸 | 🗸 |
| **Independent variable number** | 7 | 8 | 8 | 8 | 8 | 9 | 9 | 9 | 9 | 9 | 9 | 10 | 10 | 10 | 10 | 11 |
| **AIC** | 886.63 | 881.13 | 885.37 | 865.26 | 873.64 | 854.09 | 870.62 | 864.02 | 870.41 | 859.62 | 879.78 | 850.92 | 850.95 | 867.25 | 858.24 | 847.65 |
| **AICc** | 899.46 | 896.94 | 901.17 | 881.06 | 889.44 | 873.25 | 889.79 | 883.19 | 889.57 | 878.79 | 898.94 | 873.86 | 873.89 | 890.19 | 881.18 | 874.81 |
| **AICc_CMi_-AICc_CM6_** | 26.21 | 23.69 | 27.92 | 7.81 | 16.19 | 0.00 | 16.54 | 9.94 | 16.32 | 5.54 | 25.69 | 0.61 | 0.64 | 16.94 | 7.93 | 1.56 |

Note: 1) CM: candidate model; FM: final model; AIC: Akaike information criterion; AICc: second-order bias-corrected AIC, and 2) Grey cells mean variables are not included in the model.
